# Bergamot (Citrus bergamia) Essential Oil Inhalation Improves Positive Feelings in the Waiting Room of a Mental Health Treatment Center: A Pilot Study

**DOI:** 10.1002/ptr.5806

**Published:** 2017-03-24

**Authors:** Xuesheng Han, Jacob Gibson, Dennis L. Eggett, Tory L. Parker

**Affiliations:** ^1^ dōTERRA InternationalLLC, 389 South 1300 WestPleasant GroveUT84062USA; ^2^ The Green House Center for Growth and Learning135 West Center StreetPleasant GroveUT84062USA; ^3^ 223 TMCB Brigham Young UniversityProvoUT84602USA

**Keywords:** bergamot essential oil, aromatherapy, mental well‐being, mental health, positive affect, negative affect

## Abstract

Mental health issues have been increasingly recognized as public health problems globally. Their burden is projected to increase over the next several decades. Additional therapies for mental problems are in urgent need worldwide due to the limitations and costs of existing healthcare approaches. Essential oil aromatherapy can provide a cost‐effective and safe treatment for many mental problems. This pilot study observed the effects of bergamot essential oil inhalation on mental health and well‐being, as measured by the Positive and Negative Affect Scale, in a mental‐health treatment center located in Utah, USA. Fifty‐seven eligible participants (50 women, age range: 23–70 years) were included for analysis. Fifteen minutes of bergamot essential oil exposure improved participants' positive feelings compared with the control group (17% higher). Unexpectedly, more participants participated in experimental periods rather than control periods, suggesting even brief exposure to essential oil aroma may make people more willing to enroll in clinical trials. This study provides preliminary evidence of the efficacy and safety of bergamot essential oil inhalation on mental well‐being in a mental health treatment center, suggesting that bergamot essential oil aromatherapy can be an effective adjunct treatment to improve individuals' mental health and well‐being. © 2017 The Authors. Phytotherapy Research published by John Wiley & Sons Ltd.

## Introduction

Mental health disorders have been increasingly recognized as public health problems globally (Kessler *et al.*, 2015). Mental health disorders include abnormally high levels of anxiety, depression, stress, cognitive impairment, insomnia, and so on. Of note, the prevalence of stress‐related symptoms among the working class has been increasing across a wide variety of occupations (Baba *et al.*, 1999; Johnson *et al.*, 2005; McCarthy *et al.*, 2016). The health impact of mental disorders is both immediate and long term. The immediate health effects relate to overall well‐being, quality of life, work performance, and social interactions (McCarthy *et al.*, 2016). Long‐term health effects can lead to chronic diseases and premature death (Andrews and Carter, 2001). A recent World Health Organization (WHO) World Mental Health survey (*N* = 52 095) (Bruffaerts *et al.*, 2015) found a consistent association between pre‐existing mood (odds ratio = 1.3–1.4), anxiety (odds ratio = 1.2–1.7), and the subsequent onset of headaches. The WHO Global Burden of Disease Study (Bruffaerts *et al.*, 2015) estimates that mental disorders are among the most burdensome in the world, and their burden will increase over the next 4 decades. The current healthcare system has inadequately addressed individuals with mental disorders. This includes a lack of good operational propositions, lack of professionalism, low quality of care, and improper pharmaceutical prescriptions (Mercier *et al.*, 2010). Almost all prescribed drugs for mental disorders come with a long list of side effects (Jones and Lal, 1985; Bantz *et al.*, 1987; Etzel, 1994; Gerlach and Larsen, 1999; Schaefer *et al.*, 2003; Rummel‐Kluge *et al.*, 2010). Alternative therapies for mental disorders are in urgent need worldwide.

The inhalation of essential oils may provide a cost‐effective, safe, and appropriate therapy for some mental disorders. Many human studies involving a wide diversity of patients and healthy volunteers have successfully shown significant positive effects of lavender essential oil (Moss *et al.*, 2003; Toda and Morimoto, 2008; Hongratanaworakit, 2011; Seifi *et al.*, 2014) on stress relief, anxiety reduction, mood improvement, and depression relief. Chemical analysis of lavender essential oil shows a complex mixture of naturally occurring phytochemicals, including linalool and linalyl acetate (Prashar *et al.*, 2004). It has been suggested that the major components in essential oils contributing to anti‐anxiety and anti‐depressant effects are linalool (Linck *et al.*, 2010), limonene (Lima *et al.*, 2013), and pinene (Satou *et al.*, 2013). Reasonably, it has been proposed that essential oils with a high content of these compounds could have anxiolytic and anti‐depressant effects as well.

Bergamot essential oil (hereafter BEO) has a long industrial and medicinal history (Navarra *et al.*, 2015). It is characterized by a high content of limonene, linalool, and linalyl acetate. Several clinical studies on aromatherapy with BEO, in combination with other essential oils, have shown promising results: anxiety and stress reduction, anti‐depression, pain relief, and blood pressure and heart rate reduction. Further human studies with BEO inhalation alone have also shown significant effects on anxiety reduction (Watanabe *et al.*, 2015), depression reduction (Watanabe *et al.*, 2015), and blood pressure (Chang and Shen, 2011; Ni *et al.*, 2013) and heart rate reduction (Chang and Shen, 2011; Ni *et al.*, 2013). In addition, BEO has minimal side effects, if any (Navarra *et al.*, 2015), suggesting BEO inhalation may have potential therapeutic benefits including improving overall mental health and anxiety.

We explored the effect of BEO inhalation on participants' mental well‐being and feelings in the lobby of a mental health treatment center prior to receiving treatment. Participants' mental health was measured by a self‐rated questionnaire: The Positive and Negative Affect Schedule (PANAS), a standardized, well‐validated outcome measurement (Watson *et al.*, 1988). PANAS has been shown to be an effective measurement for momentary mental health and well‐being, incorporating both positive and negative dimensions of mood (Watson *et al.*, 1988; Crawford and Henry, 2004). It has also been widely used to evaluate individuals' mood states in a variety of situations (Crawford and Henry, 2004).

## Methods

This study was conducted in the waiting room of a mental health treatment center (The Green House Center for Growth and Learning, Pleasant Grove, UT, USA). Participants were current patients of the center or patients' companions. This study was reviewed and approved by an institutional review board (IRB) before commencement.

### Inclusion and exclusion criteria

Both women and men, aged between 18 and 70 years, were included if they could communicate verbally and read and write in English. Women who were pregnant and/or lactating were excluded. People with no sense of smell or with known pre‐existing sensitivities to essential oils were excluded from the study. Patients who, judged by the staff of the treatment center as not good candidates for the study, were also excluded.

### Procedure

The trial lasted 8 weeks. Weeks 1, 3, 5, and 7 were essential‐oil diffusion periods; weeks 2, 4, 6, and 8 were distilled‐water diffusion periods. Participants who met the above eligibility criteria and agreed to participate were instructed to sit as still as possible in the waiting room for 15 min. Written consent was waived per an IRB's evaluation. Depending on the week they came to the center, they were exposed to either bergamot or distilled water aromatically. Patients were told the study was intended to assess the waiting time's effect on mental feelings to distract their attention from the smell in the waiting room. After 15 min, participants were asked to complete the PANAS survey and demographic information (Fig. S1). Then, they could proceed with their regularly scheduled treatment. Any adverse events were also recorded.

### Study instruments and materials

The aromatherapy devices were four waterless diffusers, provided by dōTERRA (Pleasant Grove, UT, USA), and placed out of sight of the participants in the waiting room. The gas chromatography–mass spectrometry analysis of BEO showed that its major chemical constituents (i.e., >5%) are limonene (36%), linalyl acetate (31%), linalool (11%), gamma‐terpinene (8%), and beta‐pinene (7%).

The diffusers were turned on 15 min before the first patient arrived and kept running at half speed throughout the day. The speed setup of diffusers and their locations were selected based on previous experiments (unpublished data), ensuring that a comfortable and consistent aroma was maintained through the day. Facility staff routinely checked each diffuser to ensure there was always sufficient essential oil or distilled water, and replaced new bottles of oil or water if needed. This ensured that each participant received an equivalent strength and amount of oil aroma or water vapor during the 15‐min study period, which approximately mimicked the real‐life scenario of aromatic usage of essential oils.

The PANAS has been extensively validated and utilized to assess momentary mental well‐being (Watson *et al.*, 1988; Crawford and Henry, 2004). It consists of 20 items (e.g. ‘interested’ and ‘distressed’), and respondents rate the intensity of their feelings about each item at that moment from 1 (*very slightly or not at all*) to 5 (*extremely*). Ten items assess respondents' positive feelings, while the other ten assess negative feelings. Scores range from 10 to 50 for either positive or negative affect. The PANAS typically takes less than 5 min to complete.

### Statistical analysis

Fifty‐nine participants completed the study. Two were missing scores for one or more of the PANAS items; therefore, they were excluded from analysis. The age range was 23–58 years for the BEO group, and 23–70 years for the control group (Table 1). Most participants (50 out of 57) were female and Caucasian. Among these, only ten of them were current patients of the treatment center (the others were patients' companions).

**Table 1:** Participant's demographic information

	Active group (*n*)	Control group (*n*)
Participants	45	12
Women	38	12
Men	7	0
Women's age range (years)	23–58	23–70
Men's age range (years)	36–48	n/a
Caucasian	41	12
Non‐Caucasian	4	0
Female and Caucasian	36	12
Patients	10	0

Data were analyzed using the Statistics Analysis Software System (SAS Institute Inc., Cary, NC, USA). The analysis compared the difference between the active group (BEO) and the control group (distilled water) in positive and negative affect scores. In addition, a two‐sample *t*‐test was also performed for each individual item to compare the means for each treatment group. Statistical assessments were two tailed and considered significant at *p* < 0.05 and near significance at 0.05 ≤ *p <* 0.1.

## Results

No adverse effects were caused by exposure to BEO aroma as reported by the participants. More participants participated and completed the trial in the active group (*n* = 45) than did those in the control group (*n* = 12) (Table 1). There were ten patients in the active group and none in the control group.

Overall, the active group reported both higher positive and negative affect scores than did the control group (Table 2). The mean positive affect score for the active group was 17% higher than that of the control group, although the improvement was not considered statistically significant. The mean negative affect score for the active group was 15.4% higher than that of the control group, and the increase was not statistically significant either. A complete listing of all statistical tests is contained in Table 2.

**Table 2:** Summary of PANAS scores in the active group and the control group (*N* = 57)

	Item	Active group mean (*n* = 45)	Active group SE (*n* = 45)	Control group mean (*n* = 12)	Control group SE (*n* = 12)	Difference (active − control)	SE of difference	Degrees of freedom	*t*‐test value	*p*‐value
Positive affect	1 Interested	2.73	0.18	2.75	0.35	−0.02	0.39	55	0.04	0.97
	3 Excited	1.69	0.12	1.42	0.24	0.27	0.26	55	1.03	0.31
	5 Strong	2.60	0.18	2.33	0.36	0.27	0.31	55	0.67	0.51
	9 Enthusiastic	2.22	0.18	1.92	0.35	0.31	0.38	55	0.79	0.44
	10 Proud	2.36	0.19	1.58	0.37	0.77	0.41	55	1.87	0.07*
	12 Alert	3.13	0.16	2.67	0.30	0.47	0.34	55	1.38	0.17
	14 Inspired	2.38	0.18	1.83	0.35	0.54	0.39	55	1.39	0.17
	16 Determined	2.93	0.19	2.58	0.37	0.35	0.41	55	0.84	0.40
	17 Attentive	2.91	0.15	2.83	0.30	0.08	0.33	55	0.23	0.82
	19 Active	1.91	0.14	1.33	0.28	0.58	0.31	55	1.86	0.07*
	Total positive	24.87	1.15	21.25	2.22	3.62	2.5	55	1.45	0.15
Negative affect	2 Distressed	1.91	0.17	1.50	0.33	0.41	0.37	55	1.12	0.27
	4 Upset	1.53	0.14	1.42	0.28	0.12	0.31	55	0.38	0.71
	6 Guilty	1.38	1.14	1.50	0.27	−0.12	0.31	55	0.40	0.69
	7 Scared	1.60	0.16	1.00	0.31	0.60	0.35	55	1.70	0.10
	8 Hostile	1.16	0.08	1.33	0.16	−0.18	0.18	55	−1	0.32
	11 Irritable	1.62	0.15	1.75	0.29	−0.13	0.32	55	0.39	0.70
	13 Ashamed	1.31	0.15	1.42	0.29	−0.11	0.33	55	0.32	0.75
	15 Nervous	1.73	0.16	1.08	0.30	0.65	0.34	55	1.92	0.06*
	18 Jittery	1.60	1.50	1.33	0.29	0.27	0.33	55	0.82	0.42
	20 Afraid	1.57	0.16	1.00	0.30	0.57	0.34	55	1.67	0.10
	Total negative	15.38	1.09	13.33	2.12	2.04	2.4	55	0.86	0.40

Note: SE, standard error; PANAS, The Positive and Negative Affect Schedule.

* *p* < 0.1.

No statistically significant difference was observed for any of the individual items between the active group and control group (Table 2). However, near‐significant (0.05 < *p <* 0.1) and meaningful differences (0.4 or above on a scale from 1 to 5) were observed for several items. Participants in the active group reported a 48.77% higher score of feeling ‘proud’ compared with the control group. They also reported a 43.36% higher score of feeling ‘active.’ However, participants in the active group also reported a 60% increase of feeling ‘nervous.’

## Discussion

Participants in the waiting room of the mental health treatment center in the BEO group reported higher positive affect scores than did the control group. Participants in the BEO group also showed a smaller increase in negative affect scores than did the control group; however, the reasons why are unclear. It could be due to an effect resulting from the smaller size of the control group. Interestingly, the control group's mean negative affect score was already in the very lower end of the scale. This indicates that there was probably not much room to improve in negative affect, partially because the clear majority of participants (47 out of 57) were patients' companions, who were less likely to have mental disorders than patients. Moreover, the improvement in positive affect scores was greater than the increase in negative affect scores, although neither of them was statistically significant. Collectively, this suggests that BEO aromatherapy might provide beneficial effects on participants' mental well‐being and feelings, as measured by PANAS, in the waiting room of a mental health treatment center.

A body of clinical research has provided evidence supporting the therapeutic effects of BEO aromatherapy on mild mental disorders in a variety of settings (Navarra *et al.*, 2015). Bergamot essential oil aromatherapy provided several beneficial effects to participants including reduced heart rate, blood pressure, stress responses, depression, and anxiety (Chang and Shen, 2011; Ni *et al.*, 2013; Navarra *et al.*, 2015; Watanabe *et al.*, 2015). Although the BEO mechanism is not fully understood, some studies suggest that it may trigger the release of discrete amino acids, which then may act as neurotransmitters that interact with normal and pathological synaptic plasticity (Bagetta *et al.*, 2010; Saiyudthong and Marsden, 2011). The clinical pharmacology of BEO is out of this study's scope; however, it was recently reviewed by Mannucci and colleagues (Mannucci *et al.*, 2017).

Bergamot essential oil aromatherapy was extremely safe in this study, and no adverse events were reported. This was largely consistent with existing literature (Navarra *et al.*, 2015; Mannucci *et al.*, 2017). This might be partially true because bergamot and other citrus aromas are common; therefore, people have become used to these aromas.

To our knowledge, this study provided the first evidence of the therapeutic efficacy and safety of BEO aromatherapy in the waiting room of a mental health treatment center. Although definite conclusions of BEO's therapeutic properties remain elusive, BEO aromatherapy may play an important role in a holistic healthcare approach, especially for those dealing with issues related to mental health or well‐being (Mannucci *et al.*, 2017).

It is unclear why fewer people were willing to participate during the control periods than the BEO periods (ns = 12 vs. 45). This observation appears to be more apparent for patients of the mental health treatment center, who had already been diagnosed with mental health issues: ten patients participated in the BEO periods, while no patient participated in the control periods. To the best of our knowledge, this is unlikely due to any other interventions than the difference between exposure to BEO aroma and water vapor. One explanation would be that even very brief exposure to BEO aroma may somehow make people (specifically patients with mental issues) become more willing to participate in clinical trial in the current setting. If this is true, it can have profound applications in both clinical trial participation and compliance as well as many other scenarios.

## Limitations

This study had several limitations. It was designed as a pilot study to explore the potential benefits of BEO aromatherapy for mental well‐being and feelings. The study did not have sufficient statistical power to make definite conclusions mainly due to the small control group, small overall study size, and study design. The study design was the best possible option at the time the trial commenced. We designed it to mimic the real‐life scenarios of aroma inhalation as much as possible. Furthermore, we intended to minimize the effects that the treatment center had on the trial and its participants. Therefore, only current patients of the center and their companions were invited to participate. Further studies with designs, such as better controlling participation, are recommended.

## Supporting information

Supplementary Materials _ PANAS and Demographic Information Survey.
