# Supplementary figures and images for: Bergamot (Citrus bergamia) Essential Oil Inhalation Improves Positive Feelings in the Waiting Room of a Mental Health Treatment Center: A Pilot Study
Source: Phytother Res. 2017 Mar 24;31(5):812–6. doi: 10.1002/ptr.5806 (PMC5434918; doi:10.1002/ptr.5806)

**Supplementary Materials _ PANAS and Demographic Information Survey**


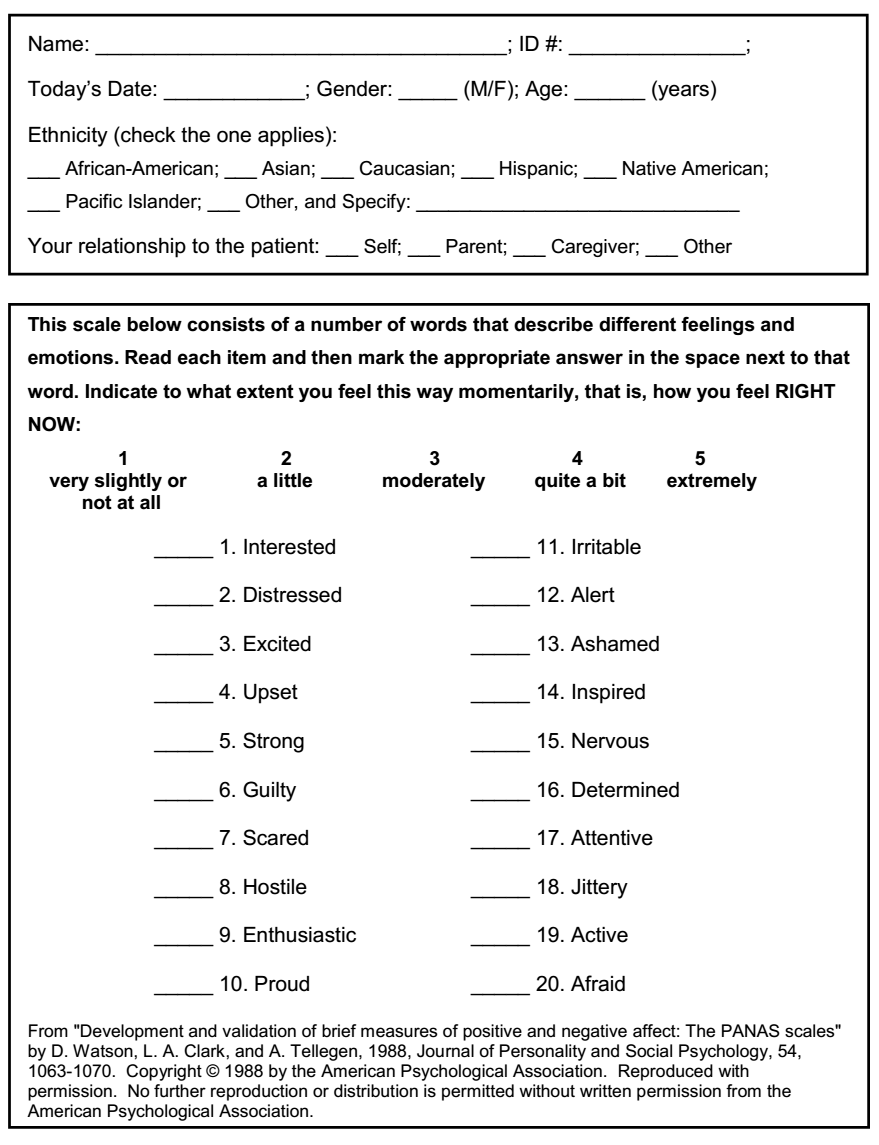

Supplement: Supplementary file 1 — Supplementary Materials _ PANAS and Demographic Information Survey. [file PTR-31-812-s001.docx]
